# *Tropilaelaps mercedesae*: an emerging global threat to apiculture – a comprehensive review

**DOI:** 10.3389/finsc.2026.1871985

**Published:** 2026-08-26

**Authors:** Mojtaba Esmaeily, Sunho Kwon, Esmaeil Babaeian, Chuleui Jung

**Affiliations:** 1 Department of Plant Medicals, GyeongKuk National University, Andong, Republic of Korea; 2 Centre for Biodiversity Genomics, University of Guelph, Guelph, ON, Canada; 3 Agricultural Research Institute, GyeongKuk National University, Andong, Republic of Korea

**Keywords:** *A. mellifera*, diagnostic methods, honey bee ectoparasitic mites, integrated pest management, *Tropilaelaps mercedesae*, Varroa destructor co-infestation

## Abstract

Honey bees (*Apis* spp.) are key pollinators in agricultural and natural ecosystems; however, their populations are declining due to multiple interacting stressors and their synergistic effects, including parasitic mites. While *Varroa destructor* is widely recognized as the primary global driver of colony losses, mites of the genus *Tropilaelaps*, particularly *Tropilaelaps mercedesae*, are emerging as a serious and still underestimated threat. Native to Asia and naturally associated with wild hosts such as *Apis dorsata*, *T. mercedesae* has successfully transitioned to managed *Apis mellifera* colonies and is now widespread across much of Asia. Recent reports from Central Asia and the Caucasus and western Eurasian regions (including Georgia and Russia) indicate that this species is undergoing an ongoing westward expansion toward Europe. Its biological traits—including an extremely short reproductive cycle, obligate dependence on sealed brood, high dispersal capacity, and the potential to transmit viruses such as deformed wing virus (DWV)—facilitate rapid population growth and severe colony-level damage, particularly in *A. mellifera*, which lacks effective behavioral defenses against this mite. This review synthesizes current knowledge on the taxonomy, morphology, life cycle, host–parasite interactions, geographic distribution, and spread of *Tropilaelaps* mites, with emphasis on *T. mercedesae*. It also evaluates available diagnostic approaches, including brood-based methods, adult bee–based methods, and natural mite-fall techniques. Furthermore, evidence on chemical and biotechnical control strategies is summarized, and their strengths, limitations, and integration within an Integrated Pest Management (IPM) framework are discussed. Overall, current findings highlight the urgent need to strengthen surveillance, standardize diagnostic protocols, and develop sustainable control strategies to prevent the global spread of *Tropilaelaps* mites.

## 1 Introduction

Honey bees (*Apis* spp.) are irreplaceable pollinators that play a crucial role in global food production and, together with other pollinators, represent one of the most important components of ecosystems, with their activity being essential for maintaining ecosystem stability (1, 2). As some of the world’s most significant pollinators, they facilitate the reproduction of flowering plants, thereby playing a decisive role in increasing agricultural yields and supporting the biodiversity of wild plant species (2). In addition to their pollination services, managed honey bees produce a wide range of hive products, including honey, bee pollen, bee bread, royal jelly, beeswax, bee venom, and propolis. These products, beyond direct human consumption, are widely used in the pharmaceutical and cosmetic industries (3) as well as in the development of biomaterials (4), demonstrating their remarkable biotechnological potential. Despite their ecological and economic importance, honey bee populations are experiencing alarming declines worldwide due to a combination of biotic and abiotic factors, including parasites, pathogens, habitat loss, pesticide exposure, and climate change (5–7). These stressors often act synergistically, intensifying colony losses and making the decline a complex, multifactorial issue (8–11). Addressing these challenges is essential to ensure the long-term sustainability of agricultural productivity and natural ecosystems.

In recent years, apiculture worldwide has faced increasing challenges as previously mentioned. Of these factors, parasitic mites represent one of the most direct and persistent threats to honey bee survival. The varroid mite, *Varroa destructor* (hereafter referred to as *Varroa*) is widely recognized as the primary cause of colony weakening and collapse across the globe (12–15). This mite feeds on the fat body and hemolymph of both adult and developing bees and transmits a wide range of viruses, leading to severe physiological and immunological impairments (16, 17). Despite the extensive damage it causes, intensive research efforts and the development of various management strategies have led to partial control of *Varroa* infestations in many regions, and investigations into improved control methods continue. Beyond *Varroa*, other parasitic mites of honey bees belonging to the genus *Tropilaelaps* have recently emerged as a growing global concern (18–20). Although their current distribution remains limited, reports of *Tropilaelaps* infestations in parts of Europe, including Georgia (21) and Russia (22), as well as in Central Asia (20) and extending to Pakistan (23), indicate a continuing westward geographic expansion. In infested regions, these mites cause significant colony losses, and when co-infestations with *Varroa* occur, the negative effects are markedly amplified (24, 25). Due to their high reproductive potential, rapid adaptability, and distinctive biological characteristics, *Tropilaelaps* mites have the potential to spread globally and become a major pest of managed honey bee colonies (18, 26–28). Therefore, comprehensive studies on the biology, ecology, and management of these mites are urgently required to prevent future apicultural crises.

The genus *Tropilaelaps* comprises ectoparasitic mites native to Asia that naturally parasitize wild honey bee species such as *Apis dorsata*, *A. laboriosa*, and *A. breviligula* (29). However, of the described species within this genus, two species— *T. mercedesae* and *T. clareae*— are capable of infesting managed colonies of both *A. mellifera* and *A. cerana* (30). The damage caused by these mites is generally more severe in the European honey bee (*A. mellifera*) due to the absence of effective behavioral defenses compared with the Asian honey bee (*A. cerana*), rendering *A. mellifera* colonies highly susceptible to parasitic infestations (31, 32). Among *Tropilaelaps* species, *T. mercedesae* is regarded as the most destructive (33). It was first identified in a colony of *A. mellifera* in Hanoi, Vietnam (Hymenoptera: Apidae) (34). Since its initial description, this species has expanded its distribution and is now reported from various regions of East and Southeast Asia. Given its broad distribution and high virulence, the present review primarily focuses on *T. mercedesae*. Therefore, in the following sections, the term *Tropilaelaps* refers specifically to this species unless otherwise stated. According to Lee et al. (35), *Varroa* infestations in South Korea were considerably higher than those of *Tropilaelaps* (91% vs. 25.7%), yet subsequent studies have revealed an increasing prevalence of *Tropilaelaps* mites in honey bee colonies (24, 28, 33). Co-infestations with both mite species have also been frequently observed, often resulting in more severe colony losses (24, 36). Like *Varroa*, *Tropilaelaps* mites can reproduce only within honey bee brood cells and, due to their specialized gnathosoma, feed exclusively on the immature stages of honey bees (26). However, their smaller body size, shorter phoretic phase, higher mobility, and rapid reproductive rate make *Tropilaelaps* an emerging and potentially more challenging threat to the beekeeping industry (18, 26–28). In addition to direct parasitic damage, these mites are also significant as vectors of viral pathogens affecting honey bees, including the deformed wing virus (DWV) (37–40). This vectoring ability, combined with their biological traits, further underscores the potential of *Tropilaelaps* as a highly dangerous pest in apiculture.

Although the current distribution of *Tropilaelaps* mites is not as extensive as that of *Varroa*, their potential spread to other continents has raised serious concerns. The predicted pattern of expansion is likely similar to *Varroa*, facilitated by international trade and colony movement (19, 20). Therefore, considering the biological characteristics and high adaptive potential of this parasite, comprehensive studies and preparedness for its effective management are essential. Given the increasing risk of global spread and the absence of standardized management protocols, an updated synthesis of *Tropilaelaps* biology, epidemiology, diagnosis, and control is urgently needed. As *T. mercedesae* is the most widespread and economically destructive species of the genus, this review primarily focuses on it; however, updated information and recent findings on *T. clareae* are also included for completeness.

## 2 Taxonomy and morphological identification

Mites of the genus *Tropilaelaps*, belonging to the order Mesostigmata and the family Laelapidae, are important ectoparasites of honey bees in Asia. Based on morphological and molecular analyses, Anderson and Morgan (34) classified this genus into four species: *T. clareae*, *T. koenigerum*, *T. mercedesae*, and *T. thaii*. Among these species, *T. koenigerum* is a parasite of *A. dorsata* in Sri Lanka, mainland Asia, and Indonesia (32, 34), whereas *T. thaii* has been observed exclusively on *A. laboriosa* in Vietnam (34). The other two species, *T. clareae* and *T. mercedesae*, have been reported on native hosts such as *A. dorsata* and *A. breviligula* (18, 34), and they have also been found parasitizing both the Asian honey bee (*A. cerana*) and the European honey bee (*A. mellifera*). They are capable of reproducing within the drone and worker brood cells of these hosts (34). However, *T. mercedesae* exhibits a much wider geographic distribution, and its detrimental impacts on managed honey bee colonies are more extensive and severe (18, 34).

*Tropilaelaps* is a small genus with not more than four recognized species and exhibits a relatively low level of morphological diversity (18). Adults are reddish-brown in color, idiosoma suboval, distinctly longer than wide, highly hypertrichous dorsally and ventrally, densely covered by short, needle-like setae; epigynal shield narrow, very elongate, abutting or overlapping the subquadrate anal shield; corniculi reduced or membranous; cheliceral digits short, stumpy with reduced teeth; tarsi with well-developed ambulacral pads but with reduced claws (41, 42). The dentition of the chelicerae appears to be specialized, and is thought to be an adaptation for creating feeding wounds in the integument and for a parasitic lifestyle (43). Leg I is distinctly more slender than legs II–IV. Leg setae generally long, with some setae thickened (44). The nymphal stages are whitish in color, and like the adults, their idiosoma is narrow and longer than wide, a feature that together with slender legs, enables the mites to move rapidly across the bee’s body surface. Morphological identification of *Tropilaelaps* species remains challenging, primarily due to brief and incomplete original descriptions and a wide range of morphological variation. In addition, some important character states such as leg and palp chaetotaxy, gnathosomal structures, pores and poroids have largely been overlooked. Comprehensive modern redescriptions of these species remain limited; therefore, careful examination of type specimens is required for accurate species delimitation.

Oldroyd and Wongsiri (45) distinguished two species *T*. *clareae* and *T*. *koenigerum* based only on the shape of the anal shield (pear-shaped versus horseshoe-shaped). However, we know that anal shield morphology can show intra-specific or artificial variation due to differences in the degree of sclerotization of the shield, particularly when larger series of specimens are examined. Consequently, this character alone may be insufficient for reliable species-level diagnosis.

Anderson and Morgan (34) examined species diversity in *Tropilaelaps* by analyzing cytochrome oxidase I (COI) and nuclear ribosomal DNA (nrDNA) genes from specimens collected from different honey bee hosts across Asia and surrounding regions. Their findings indicated the presence of at least four distinct species in the genus *Tropilaelaps*, demonstrating that species diversity in the genus has been underestimated and that morphology alone can be insufficient for reliable species identification. This comparison shows that the four groups (Clades 1–3 and Taxon A) are differentiated primarily by a combination of body size, the shape of the posterior margin of epigynial, anal shield, the dentition of the movable digit, with male spermatodactyl character providing additional support where available.

In addition to the limited and often overlapping interspecific morphological differences among *Tropilaelaps* species, increasing evidence indicates substantial intraspecific morphological variation within *T. mercedesae* populations across different geographic regions. Comparative studies have documented variation in several diagnostic traits, including ventral shield sclerotization, opisthogastric setation, and cheliceral morphology. Based on currently available molecular evidence, these differences are more likely to represent geographically structured intraspecific variation rather than species-level divergence. Such variability further complicates morphology-based identification and may partially explain inconsistencies among previous taxonomic descriptions. Given that the original description of *T. mercedesae* lacks detailed information on several important characters, including leg chaetotaxy and fine gnathosomal structures, these findings highlight the need for a comprehensive morphological revision based on geographically diverse populations (20).

## 3 Life cycle, survival, and dispersal

**Figure 1:**
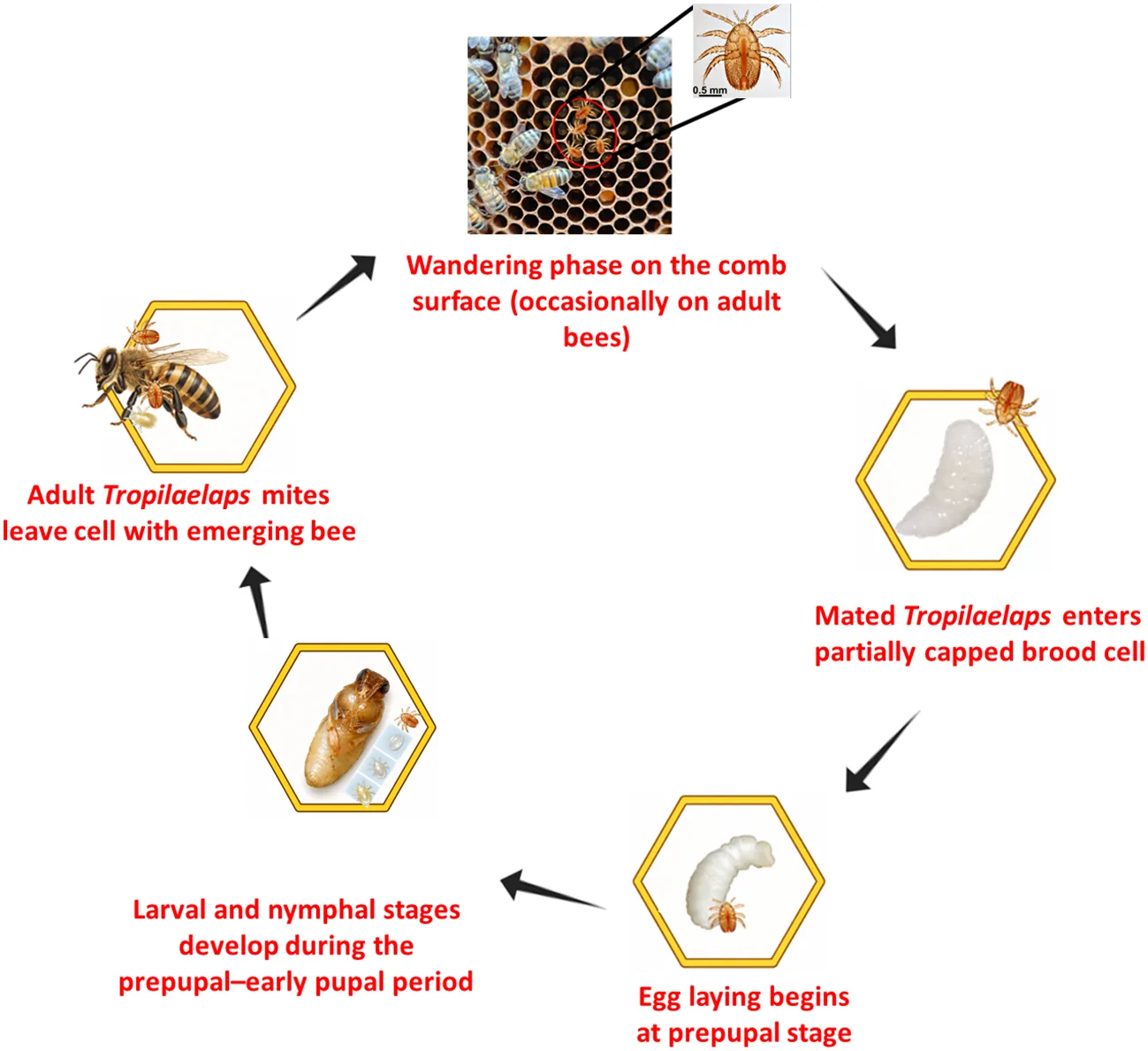
Life cycle of the parasitic mite *T. mercedesae*. A mated female enters a partially capped brood cell, lays eggs during the prepupal stage, and offspring develop through larval, protonymphal, and deutonymphal stages within the sealed cell. Adult mites emerge with the host bee and survive briefly on combs or adult bees before invading a new brood cell. The honey bee pre-adult developmental images (larva, prepupa, and pupa) were adapted from developmental images available at Scientific Beekeeping (http://scientificbeekeeping.co.uk/genPupae.html).

The life cycle of *Tropilaelaps*, which is closely associated with honey bee brood development, is summarized in **Figure 1**. It begins when a mated adult female enters a partially capped brood cell containing a late-stage larva (46, 47). Typically, only one foundress infests a given cell (46). Unlike *Varroa*, feeding on the larva is not required to initiate oviposition in *Tropilaelaps* (48). Females become gravid about two days after mating, and egg-laying generally occurs once the host larva reaches the prepupal stage (48, 49). Fecundity varies with the host species: in *A. mellifera* it averages around 1.4 offspring, whereas in *A. dorsata*, it can reach approximately 2 offspring, although colony conditions and environmental factors may cause slight variation (18, 28, 46). The sex ratio is consistently female-biased and ranges from 1:2 to 1:56 depending on host species and environmental conditions. This pattern has been attributed to haplodiploid sex determination, shorter male longevity, and the tendency for female offspring to be produced first in the oviposition sequence (41, 46, 50, 51).

After hatching, the developing mite passes through a larval stage followed by two nymphal stages (protonymph and deutonymph), before molting into the adult stage (43). Development within *A. mellifera* worker cells typically takes 6–9 days (47, 49), with males maturing approximately 24 hours before females (41). Shortly before adult bee emergence, late-stage deutonymphs are usually present, and most mites have completed development by this time (18).

Following emergence from the brood cell, adult mites typically survive for only 1–2 days on adult bees, resulting in a brief phoretic period and a strong dependence on the continuous presence of brood (52). Morphological traits such as an elongated body and enlarged ambulacra facilitate efficient attachment and movement on the host (41). Dispersal can occur via drifting bees, robbing behavior, or through flower-mediated transfer between infested and non-infested pollinators, potentially enabling movement between colonies and, under certain circumstances, between host species (46, 53).

## 4 Host–parasite interaction

Mites of the genus *Tropilaelaps* are primarily parasites of giant Asian honey bees, particularly *A. dorsata*, as well as *A. laboriosa* and *A. breviligula*, and have evolved as integral components of the natural ecosystems of these hosts (26, 28, 32, 34). Despite existing limitations in the available evidence, current evidence suggests a long history of coevolution between *Tropilaelaps* mites and their native hosts, which may have contributed to the establishment of a relatively stable host-parasite association. This equilibrium is maintained through a suite of biological and behavioral traits of native Asian honey bees. These mechanisms vary in composition and intensity among species and populations and include seasonal migration, frequent colony absconding, the natural occurrence of broodless periods, and effective defensive behaviors such as grooming and hygienic removal of infested brood. Collectively, they constrain rapid mite population growth and prevent severe colony-level damage.

The host shift of *Tropilaelaps* mites to managed honey bees, including both *A. mellifera* and *A. cerana*, represents a relatively recent phenomenon on an evolutionary timescale and is strongly influenced by human-mediated changes in apiculture. The introduction and intensive management of *A. mellifera* in Asia have created novel ecological conditions that facilitate host expansion, including high colony densities, prolonged brood availability throughout much of the year, reduced natural interruptions of mite reproduction, and frequent contact between native and managed honey bee colonies (18). Among *Tropilaelaps* species, *T. clareae* and *T. mercedesae* have successfully adapted to *A. mellifera* and are capable of reproducing within both drone and worker brood cells of this novel host; however, *T. mercedesae* has proven to be markedly more successful and destructive, exhibiting a broader geographic distribution, reported haplotypic diversity, and substantially greater colony-level impact (27, 34).

In contrast to native hosts, *A. mellifera* generally lacks the highly effective co-evolved defenses observed in native Asian honey bee hosts. This evolutionary mismatch links the absence of such defenses to rapid mite reproduction, elevated infestation levels, and severe pathological effects at both the brood and colony scales (54–56). This transition from a long-standing co-evolved association to a maladapted host–parasite interaction provides a useful framework for understanding the pronounced virulence of *T. mercedesae* in *A. mellifera* colonies and sets the stage for examining the biological, behavioral, and disease-related consequences of infestation discussed in the following sections.

Across its expanded host range, *T. mercedesae* exhibits pronounced spatial, seasonal, and host-dependent variation in infestation intensity and associated damage. Field studies have consistently shown that infestation levels and colony outcomes differ markedly among regions and management systems, reflecting the combined influence of host biology, colony demography, and environmental conditions (23, 27, 28, 57). In managed *A. mellifera* colonies, where brood is often continuously available and natural regulatory mechanisms are limited, these conditions favor rapid mite population growth and the accumulation of pathological effects. Under high infestation pressure, *T. mercedesae* can drive colonies beyond their capacity to compensate for brood losses, and colony collapse has been reported in multiple regions where infestation levels remain uncontrolled (19).

Similar to *Varroa*, *T. mercedesae* shares a comparable life cycle and feeding requirement, consuming the hemolymph of honey bee larvae and pupae and causing severe developmental damage (18, 19). Due to intrinsic biological traits, particularly its shorter reproductive cycle, *T. mercedesae* is more prevalent than *Varroa* in several Asian countries, including Thailand (19), and has been reported to cause more serious colony-level damage in China (28). Its parasitism can weaken or kill brood (18, 58, 59), reduce the weight and longevity of adult bees (58, 60), induce deformities or mortality in newly emerged workers (61), and lower total protein concentration in pupae (62). Infestation also decreases honey production (59, 63), increases colony susceptibility to secondary pests such as wax moths (64), and may ultimately result in colony death (63, 65). In some heavily infested colonies, a “bald brood” pattern—where worker bees remove the cell cappings—has been observed (27). More recently, it has been shown that *T. mercedesae* infestation negatively affects key behaviors such as olfactory performance, flight ability, and homing success, and alters the expression of genes involved in immunity, carbohydrate transport, and metabolic processes (66).

In addition to the direct detrimental effects of *T. mercedesae* on honey bee brood—which alone can substantially weaken colonies—virus transmission represents another critical dimension of infestation that may further exacerbate colony decline. Although the role of *T. mercedesae* as a vector of honey bee viruses has not yet been fully clarified, accumulating evidence indicates that this mite can contribute to the transmission and amplification of several viral pathogens. Deformed wing virus (DWV) and black queen cell virus (BQCV) have been detected in honey bees parasitized by *T. mercedesae* (39), and significantly higher DWV prevalence and viral titers have been documented in infested brood (37, 39, 59). The presence of high levels of DWV genomic RNA in the transcriptome of *T. mercedesae* (67), together with the detection of negative-strand DWV RNA in the mite (68), indicates that viral replication may occur in this parasite. Moreover, Yang et al. (69), using an infectious DWV-GFP clone and RT-qPCR, demonstrated that viral loads in *T. mercedesae* were higher than those in *A. mellifera*, supporting the potential role of this mite in DWV transmission. Nevertheless, compared with *Varroa*, the capacity of *T. mercedesae* to transmit DWV remains less well characterized and requires further investigation. Additionally, the first evidence of acute bee paralysis virus (ABPV) in both *A. cerana* and *T. mercedesae* in northern Thailand (70) highlights the need for more comprehensive studies on mite–virus interactions. Beyond direct viral transmission, the multiple feeding-induced puncture wounds caused by this mite may facilitate mechanical entry of viruses and other pathogens into developing brood (19). Overall, available evidence suggests that *T. mercedesae* may contribute to the transmission of honey bee viruses through a combination of feeding damage, tissue injury, and potential virus-associated interactions. However, its role as a confirmed biological vector remains incompletely understood and requires further investigation.

## 5 Geographic distribution and spread

The genus *Tropilaelaps* (Acari: Laelapidae) is native to continental Asia and was originally associated with the giant Asian honey bee (*A. dorsata*). The genus was first described by Delfinado and Baker (71), who reported *T. clareae* from a colony of the European honey bee (*A. mellifera*) and from nest-building field rats located near the same hive in the Philippines. Subsequent research by Laigo and Morse (64) demonstrated that the natural and primary host of *Tropilaelaps* mites is *A. dorsata*.

**Figure 2:**
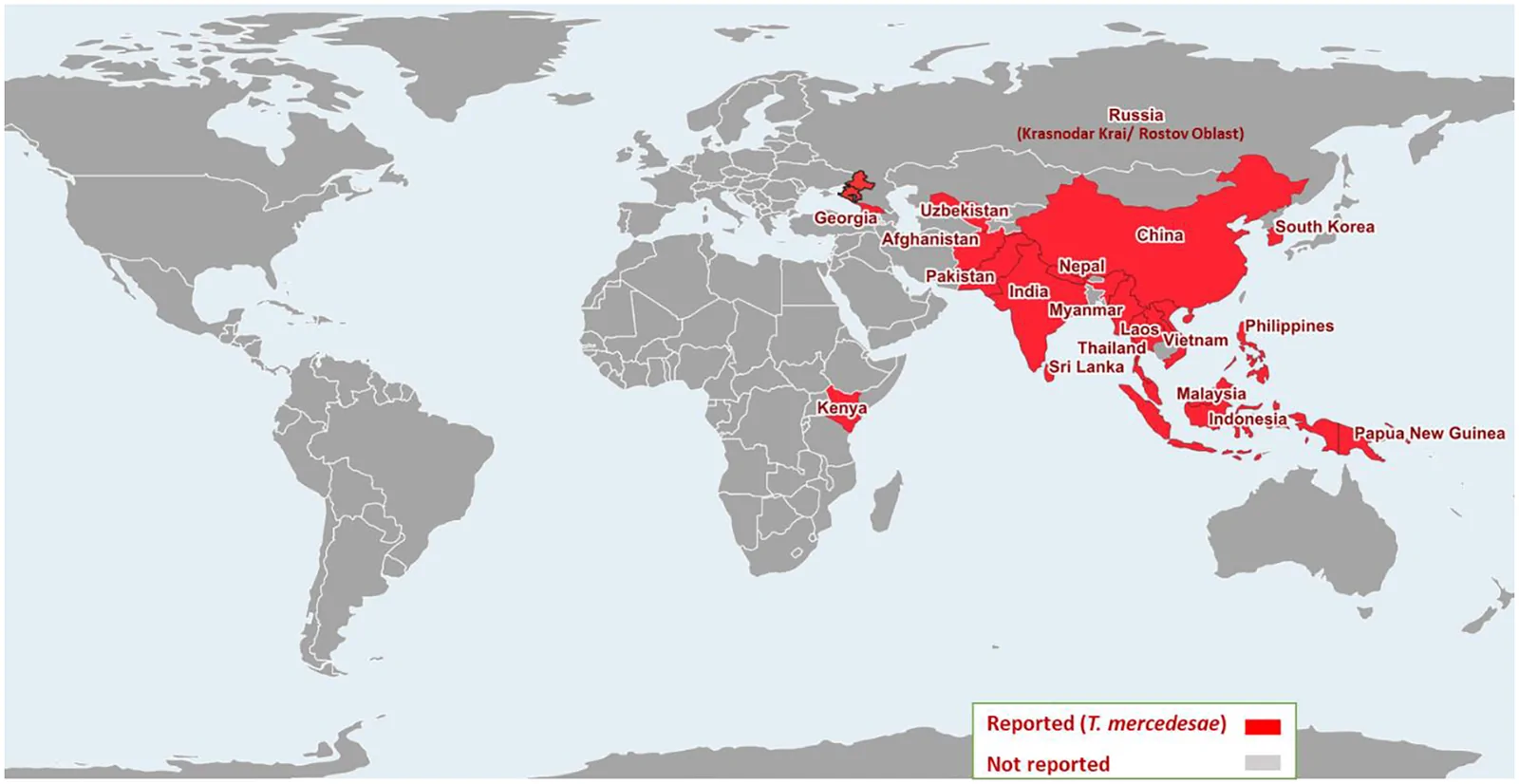
Global distribution of *T. mercedesae* based on published records. Countries highlighted in red indicate reported presence, while grey areas represent regions with no confirmed reports to date.

Therefore, the biogeography of *Tropilaelaps* mites largely mirrors the natural distribution of *A. dorsata* (34). Both *Tropilaelaps* and its native host, *A. dorsata*, are widely distributed across mainland Asia, including the Palawan Islands of the Philippines (34). Among all species of the genus, *T. mercedesae* is the most widespread and economically important. This mite primarily parasitizes *A. dorsata* throughout continental Asia and the Indonesian archipelago and has also been reported from Sri Lanka and the Palawan Islands of the Philippines. In addition to its native host, *T. mercedesae* is capable of infesting and reproducing in colonies of *A. mellifera*. Consequently, *T. mercedesae* has been reported from numerous countries, including Afghanistan (72), China (34), India (34, 73–76), Indonesia (34), Kenya (30), Laos (34), Malaysia (34, 77), Myanmar (32, 34), Nepal (29, 34), Pakistan (32, 63), the Philippines (34), Papua New Guinea (34), South Korea (34), Sri Lanka (34), Thailand (34), and Vietnam (34, 78). Subsequent reports have confirmed the extensive distribution of this species across much of Asia and its continuing spread into new regions (20, 23). Like *Varroa*, *T. mercedesae* has extended beyond its original host range and has been successfully established in regions where *A. dorsata* is absent, highlighting its ecological plasticity and potential for host switching. In recent years, *T. mercedesae* has also been recorded from Central Asia, Uzbekistan (20), where it is now considered a major emerging pest in nearly all beekeeping regions. Although no confirmed records currently exist for western Asian countries such as Iran, Turkey, and Azerbaijan, the recent westward expansion of *T. mercedesae* highlights the potential risk of future establishment in these regions. Furthermore, recent detections in Georgia (21) and Russia (22, 79) indicate that *T. mercedesae* is spreading westward across Eurasia, with a high likelihood of further expansion into Europe. An overview of the reported global distribution of *T. mercedesae*, encompassing both its native range and subsequent spread beyond the distribution of *A. dorsata*, is presented in **Figure 2**. Collectively, these findings demonstrate that *Tropilaelaps* mites—particularly *T. mercedesae*—are undergoing a progressive range expansion and are likely to be reported from additional countries in the near future.

## 6 Diagnostic methods

Continuous and accurate monitoring of mite populations is essential for their effective management. Existing diagnostic approaches can be broadly categorized into four groups: brood-based examination methods, adult bee–based methods, and techniques that rely on recording natural mite fall (27, 80). Among these, brood-based approaches generally provide the highest diagnostic sensitivity, as mites complete nearly their entire life cycle within sealed brood cells; however, the other methods also offer specific advantages depending on the monitoring objective and field conditions.

### 6.1 Brood-based diagnostic methods

These approaches rely on opening sealed brood cells and directly examining the larval or pupal stages for the presence of mites. Early techniques, such as uncapping brood with an uncapping fork—originally adapted from *Varroa* monitoring—showed limited sensitivity for detecting *Tropilaelaps*. This limitation is largely due to damage to pupae and the presence of sticky cell contents, which can obscure mites and allow them to remain hidden along the cell walls (81). To address these limitations, a more precise technique using fine forceps was introduced, in which each brood cell is individually opened and its contents carefully inspected. According to Gill et al. (82), this method exhibited the highest diagnostic sensitivity among the methods evaluated and reliably detected even low-level infestations. Other approaches, such as the bump test—where brood frames are struck to dislodge mites—are faster but exhibit only moderate sensitivity in multiple studies and may cause physical damage to brood (27). Nevertheless, recent field evaluations reported a diagnostic sensitivity of approximately 73% for the brood frame bump test, placing it among the more effective rapid-monitoring approaches and providing a favorable balance between sensitivity and operational efficiency (83). Additionally, an innovative technique known as rapid brood decapping, which employs cosmetic wax strips to quickly remove cappings followed by short video recording of emerging mites, has been shown to markedly reduce sampling time while achieving approximately 90% concordance with traditional brood-examination methods (84). Overall, while brood-based methods differ in speed and level of destructiveness, recent evidence indicates that precise cell-by-cell uncapping using forceps and the newer rapid decapping technique offer the highest sensitivity and reliability for monitoring *Tropilaelaps* infestations.

### 6.2 Adult bee–based diagnostic methods

Adult bee–based diagnostic methods involve sampling worker bees and dislodging phoretic mites using mechanical or chemical techniques. Although these approaches are widely used for monitoring *Varroa*, their effectiveness for *Tropilaelaps* is inherently limited because this mite has a very short phoretic phase and remains predominantly within brood cells. Nevertheless, some of these methods—particularly the powdered sugar shake—remain valuable tools for preliminary monitoring due to their non-destructive nature and ease of use (82). Common techniques in this category include alcohol washing, powdered sugar shaking, and CO_2_ anesthesia. Alcohol washing, which is considered the “gold standard” for *Varroa* monitoring, efficiently removes mites attached to adult bees. However, multiple studies have shown that its diagnostic sensitivity for *Tropilaelaps* is low, as these mites do not remain on adult bees long enough to be reliably recovered (27, 81). CO_2_ anesthesia has also been evaluated as a non-destructive alternative, but its sensitivity has proven insufficient, and it has consistently performed less effectively than other methods (82). Among these methods, the powdered sugar shake has been reported to provide the best relative performance in some comparative evaluations (82). This technique works by coating bees with powdered sugar and stimulating grooming behavior, which dislodges mites. Although its sensitivity remains limited, especially under low infestation levels, its speed, simplicity, and non-destructive nature make it an acceptable and practical option for beekeepers (27, 82). However, more recent studies have suggested that both powdered sugar shake and alcohol wash exhibit substantially lower sensitivity than brood-based methods and sticky-board monitoring for the detection of *T. mercedesae* (83).

### 6.3 Natural mite–fall–based diagnostic methods

Natural mite–fall approaches rely on collecting mites that naturally detach from adult bees or brood and fall onto a trapping surface placed at the bottom of the hive. These methods are widely used for monitoring *Varroa* (85, 86) and have more recently been evaluated for the surveillance of *Tropilaelaps* mites as well (27, 82). The most common tool in this category is the sticky board, which is typically placed beneath a mesh floor to prevent bees from contacting the adhesive surface. Mites that fall naturally—due to movement, grooming behavior, or weakened attachment—adhere to the board and are subsequently counted to provide a colony-level estimate of infestation intensity. Although natural mite-fall is a non-destructive and colony-wide monitoring method, its sensitivity can vary depending on colony conditions, infestation level, sampling duration, and the type of trapping surface used. This variability reflects the biology of *Tropilaelaps*, including its short phoretic phase and predominant association with brood cells. Nevertheless, recent field evaluations have shown that sticky boards can outperform several adult bee–based methods and, under certain conditions, detect *Tropilaelaps* infestations more reliably than alcohol washing or CO _2_ anesthesia (82). In a comparative study of five monitoring methods, bottom-board sticky traps achieved the highest diagnostic sensitivity (100%), exceeding worker brood uncapping (92.3%), brood frame bump tests (73.1%), alcohol wash (38.5%), and powdered sugar shake (34.6%) (83). While this method cannot provide an immediate estimate of infestation levels, it remains a valuable complementary tool—particularly for non-destructive monitoring or colony-level screening. When used alongside brood-examination techniques or powdered sugar shaking, natural mite–fall monitoring can substantially enhance the likelihood of early detection in colonies at risk of *Tropilaelaps* introduction (81, 82).

### 6.4 Environmental DNA–based method

Recent advances in molecular diagnostics have established environmental DNA (eDNA)–based approaches as powerful tools for the early detection of pest and pathogen infestations in agricultural and forest systems (87–91). In this context, eDNA offers a promising strategy for the early detection of *Tropilaelaps* infestations. Unlike traditional diagnostic methods that require direct observation of mites within brood cells or on adult bees, eDNA techniques detect trace amounts of mite DNA left in the hive environment, enabling indirect identification of infestations even when mites are not visually observed. In this approach, DNA traces originating from mites—such as fragments shed from bodies, fecal material, or degraded tissues—are collected from hive surfaces or associated materials and subsequently analyzed using PCR-based assays. Aurell et al. (92) demonstrated that eDNA extracted from hive-related substrates can be successfully used to detect *Tropilaelaps* DNA, providing a sensitive and scalable diagnostic tool for surveillance programs. In their study, several sample types were evaluated, including entrance swabs, bottom board debris, and adult bee samples. Among these, entrance swabs showed particularly high diagnostic sensitivity, likely because mites moving across the comb surface or exiting brood cells frequently contact hive entrances, leaving detectable DNA traces. The authors also reported that eDNA detection sensitivity varied among sample types. Entrance swabs consistently yielded the strongest detection signals, whereas bottom board debris and adult bee samples showed lower but still detectable levels of mite DNA. Because sampling involves simple swabbing or collection of hive debris rather than destructive brood examination, the method is well suited for large-scale monitoring programs and for surveillance in regions where *Tropilaelaps* mites are not yet established. Despite its considerable promise, eDNA-based surveillance also has several practical limitations. Sample collection still requires visits to apiaries, and subsequent analyses depend on molecular laboratory infrastructure, specialized equipment, and trained personnel. In addition, costs associated with DNA extraction and PCR-based detection may currently exceed those of conventional field-based diagnostic methods, potentially limiting routine application in some beekeeping systems. Therefore, eDNA approaches are best viewed as complementary tools rather than replacements for established diagnostic techniques. Overall, eDNA-based diagnostics represent a promising complementary approach for *Tropilaelaps* surveillance. While further validation under diverse field conditions is still required, this method offers several advantages, including non-destructive sampling, rapid processing, and the potential for early detection of infestations before visible symptoms or high mite populations occur (92).

## 7 Control and management strategies

### 7.1 Chemical and chemical-derived control strategies

Despite well-recognized limitations—including residue accumulation in hive products, adverse effects on honey bees and the environment, and the development of mite resistance—chemical control remains the most widely used and accessible approach for managing *Tropilaelaps* mites. Although no product has been specifically approved for *Tropilaelaps* control, and no dedicated veterinary medicinal products are currently available for this mite, the acaricides routinely used against *Varroa*—including amitraz, coumaphos, fluvalinate, and flumethrin—have demonstrated efficacy against *Tropilaelaps* and are commonly applied in regions where these mites are prevalent (18, 63, 93–96). Sulfur has also been reported as an effective control agent in several areas (97). Nevertheless, all these synthetic acaricides have repeatedly been shown to exert negative impacts on different aspects of honey bee health, ranging from sublethal effects to colony-level impairment, and this has been documented extensively (97–100). Such concerns highlight the urgent need for safer and more sustainable alternatives.

Alongside conventional acaricides, organic acids—primarily formic and oxalic acid—and plant-derived compounds such as thymol and hop extracts, which are widely used against *Varroa*, have also shown potential for *Tropilaelaps* management (18). For instance, Pettis et al. (97) demonstrated that formic-acid–based treatments significantly reduced mite populations up to 8 weeks’ post-treatment, and Alam et al. (94) reported formic acid achieving up to 95% efficacy, surpassing fluvalinate. The strong performance of formic acid has been further confirmed in multiple additional studies (101, 102). In a seasonal evaluation of acaricides in Korean colonies, Oh (103) found that oxalic acid (fumigation) and amitraz were most effective during spring, whereas formic acid was the most effective treatment when capped brood was present, providing simultaneous control of both *Varroa* and *Tropilaelaps*. Fluvalinate consistently underperformed across all seasons, producing minimal reduction in mite populations.

Other plant-derived compounds—including thymol and limonene (104), tobacco smoke (26), and lemongrass oil (105)—have also shown acaricidal potential. However, while essential oils frequently produce high mite mortality under laboratory conditions, they have repeatedly failed to deliver reliable efficacy under field conditions, and both application methods and effective concentrations require further optimization (106). Importantly, although organic acids and botanical extracts are generally considered safer than synthetic acaricides, their use is not without limitations. Their efficacy is highly variable across studies and strongly influenced by environmental, colony, and brood-rearing conditions. Moreover, several negative effects on honey bees—including reduced brood production, behavioral disruption, and sublethal physiological stress—have also been documented (18, 107–111). Taken together, these findings indicate that chemical control—whether synthetic or organic—cannot be solely relied upon for sustainable management of *Tropilaelaps*. Instead, chemical treatments should be integrated with complementary non-chemical strategies within an Integrated Pest Management (IPM) framework to achieve long-term, effective, and bee-safe mite control.

### 7.2 Biotechnical/beekeeping methods

Biotechnical control approaches developed for *T. mercedesae* are grounded directly in the biology and life cycle of the mite. This species completes nearly its entire reproductive cycle inside capped brood cells of honey bees and spends only a very short period—typically 1 to 3 days—on adult bees during its phoretic phase (18, 72, 112). Because *T. mercedesae* is strictly dependent on developing larvae and cannot survive or reproduce in the absence of suitable brood (34, 55), any intervention that creates a temporary broodless period or limits access to brood effectively disrupts its reproductive cycle and leads to rapid starvation of phoretic mites. This strict dependence on capped brood for reproduction has made strategies such as brood interruption and colony splitting among the most effective non-chemical tools for managing this pest. Inducing a broodless period by caging the queen for 21–24 days is one of the most extensively studied methods for fully interrupting the mite’s reproductive cycle, as remaining phoretic mites experience high mortality within a short period (113). Similarly, colony division and nucleus creation naturally generate a broodless window in both the parent and split colonies, resulting in a significant reduction in *T. mercedesae* populations—an effect demonstrated clearly in field studies in Thailand (97). However, as with chemical treatments, biotechnical methods do not consistently achieve complete control of *T. mercedesae* when applied as stand-alone interventions. Their effectiveness is strongly influenced by colony condition, season, and brood availability, and in many cases, sustainable suppression of mite populations can only be achieved through the coordinated application of multiple management approaches, underscoring the need for an Integrated Pest Management (IPM) framework.

### 7.3 Integrated pest management strategies

Effective and sustainable management of *T. mercedesae* requires the implementation of an Integrated Pest Management (IPM) framework in which biotechnical methods and chemical and chemical-derived treatments—including organic acids and plant-derived compounds—are applied in a coordinated and strategic manner rather than as stand-alone interventions. The biological characteristics of this mite, particularly its extremely short phoretic phase and strict dependence on capped brood for reproduction, severely limit the effectiveness of routine, single-measure control approaches and emphasize the need for precise timing of interventions and colony-specific decision-making. Within an IPM framework, brood manipulation functions as a foundational component by temporarily interrupting mite reproduction, thereby reducing population pressure and creating favorable conditions for subsequent interventions. Under these circumstances, the targeted and time-restricted application of chemical and chemical-derived treatments—such as organic acids—can be used to suppress residual phoretic mite populations without imposing excessive selection pressure or cumulative stress on the colony. Empirical evidence strongly supports this integrative approach. For example, Tokach et al. (114) demonstrated that combining a brood break with registered treatments based on formic acid (FormicPro^®^) or oxalic acid dribble (Api-Bioxal^®^) resulted in highly effective suppression of *T. mercedesae* infestations. Whereas mite infestation levels in untreated control colonies increased from 0.4% to 15.25% over a 60-day period, all integrated treatments evaluated in that study maintained infestation levels below 0.11%. Nevertheless, the observation of slight reductions in adult bee populations in some treatment combinations—particularly brood interruption combined with formic acid—indicates that even integrated strategies must be applied cautiously, with careful consideration of season, colony strength, and brood dynamics.

Overall, IPM enables a reduction in both the frequency and intensity of chemical and chemical-derived interventions, thereby lowering the risk of acaricide resistance, sublethal effects, and chronic colony stress. Although confirmed cases of acaricide resistance in *T. mercedesae* remain limited, experience from *Varroa* management suggests that excessive dependence on chemical treatments may favor the emergence of resistant mite populations. Therefore, resistance management should be considered an important component of future IPM programs. In addition, the practical implementation of IPM may differ substantially among regions and beekeeping systems. Climatic conditions strongly influence brood availability, colony development, and treatment efficacy, while differences in colony management practices, labor availability, and access to monitoring tools may affect the feasibility of specific IPM interventions. Consequently, management strategies that are effective in one region may require adaptation before being applied under different environmental or apicultural conditions. Nevertheless, the effective implementation of IPM remains constrained by several critical gaps, including the absence of standardized infestation thresholds, limited region-specific management guidelines, and the lack of control agents developed explicitly for this mite. Addressing these limitations through coordinated research efforts, the development of regionally adapted IPM protocols, and the integration of mite biology with practical beekeeping realities will be essential for achieving long-term, sustainable control of *T. mercedesae* in both endemic and newly invaded regions.

## 8 Conclusion and outlooks

*Tropilaelaps* mites, and particularly *T. mercedesae*, have moved from being a regionally important parasite of Asian honey bees to an emerging global threat to apiculture. Their biology—short reproductive cycle, strong dependence on brood, high mobility, and potential capacity to transmit key viral pathogens—means that even moderate infestations can rapidly escalate into serious colony-level damage, especially in *A. mellifera* populations that lack effective behavioral defences. The recent expansion of *T. mercedesae* beyond its native host range and geographic distribution, together with increasing reports of co-infestation with *Varroa*, clearly indicates that this mite can no longer be regarded as a local or secondary problem.

At present, our knowledge of *T. mercedesae* biology, life history, and host–parasite interactions provides a solid foundation for understanding its success as a parasite, but remains inadequate for fully predicting or preventing its further spread. Critical aspects of its ecology—such as survival and dispersal dynamics under different climatic conditions, interactions with local bee subspecies, and the quantitative contribution of this mite to virus transmission—remain incompletely understood. Likewise, although molecular and morphological tools now allow reliable discrimination between *T. mercedesae* and *T. clareae*, there is still limited information on intraspecific variation, local adaptation, and the potential for rapid evolutionary responses under control pressure. These knowledge gaps limit our ability to develop robust risk-assessment models and to design targeted, evidence-based management strategies.

In terms of management, most current control strategies are adapted from *Varroa* control programs and provide only partial suppression of *Tropilaelaps* infestations. The biological differences between these mites highlight the need for more targeted approaches and further optimization of existing control tools, particularly within integrated management frameworks. Ultimately, the continued westward expansion of *T. mercedesae* across Eurasia underscores the urgent need for coordinated international action. The global spread of *Varroa* has already demonstrated how rapidly a parasitic mite can become established worldwide once it enters the commercial network of managed colonies. Therefore, countries that remain free of *Tropilaelaps* should not assume long-term security, but instead implement proactive preventive measures and targeted research without delay. Strengthening sanitary and veterinary regulations, expanding surveillance programs in high-risk regions, establishing harmonized reporting systems, and providing practical training for beekeepers are essential for early detection and for slowing the rate of spread. Under these circumstances, *Tropilaelaps* should be regarded not as a hypothetical future risk, but as an imminent threat. Proactive mitigation—through rigorous research, standardized monitoring, and integrated management—will be critical for safeguarding honey bee health, sustaining pollination services, and preserving the stability of agricultural and natural ecosystems in the decades to come.
